# Camel Hemorphins Exhibit a More Potent Angiotensin-I Converting Enzyme Inhibitory Activity than Other Mammalian Hemorphins: An In Silico and In Vitro Study

**DOI:** 10.3390/biom10030486

**Published:** 2020-03-23

**Authors:** Amanat Ali, Seham Abdullah Rashed Alzeyoudi, Shamma Abdulla Almutawa, Alya Nasir Alnajjar, Yusra Al Dhaheri, Ranjit Vijayan

**Affiliations:** Department of Biology, College of Science, United Arab Emirates University, PO Box 15551 Al Ain, Abu Dhabi, UAE

**Keywords:** hemorphins, angiotensin-I converting enzyme, anti-hypertension, molecular docking, molecular dynamics

## Abstract

Angiotensin-I converting enzyme (ACE) is a zinc metallopeptidase that has an important role in regulating the renin-angiotensin-aldosterone system (RAAS). It is also an important drug target for the management of cardiovascular diseases. Hemorphins are endogenous peptides that are produced by proteolytic cleavage of beta hemoglobin. A number of studies have reported various therapeutic activities of hemorphins. Previous reports have shown antihypertensive action of hemorphins via the inhibition of ACE. The sequence of hemorphins is highly conserved among mammals, except in camels, which harbors a unique Q>R variation in the peptide. Here, we studied the ACE inhibitory activity of camel hemorphins (LVVYPWTRRF and YPWTRRF) and non-camel hemorphins (LVVYPWTQRF and YPWTQRF). Computational methods were used to determine the most likely binding pose and binding affinity of both camel and non-camel hemorphins within the active site of ACE. Molecular dynamics simulations showed that the peptides interacted with critical residues in the active site of ACE. Notably, camel hemorphins showed higher binding affinity and sustained interactions with all three subsites of the ACE active site. An in vitro ACE inhibition assay showed that the IC_50_ of camel hemorphins were significantly lower than the IC_50_ of non-camel hemorphins.

## 1. Introduction

Hypertension is a significant global health problem and its timely detection and management is essential in reducing the mortality rate. Globally, hypertension is associated with approximately 13.5% of all deaths [1]. Recent studies suggest that approximately 972 million adults have hypertension, and this number is projected to increase to 1.56 billion by 2025 [2]. Hypertension is considered to be a major risk factor for heart related disorders and it is responsible for over nine-million deaths each year [3]. The pathophysiology of hypertension is highly complex and multiple risk factors are involved. Among these risk factors stress, genetics, smoking, high sodium and alcohol intake, obesity, and environmental factors are well characterized and they are strongly associated with hypertension. In addition to these, the hyperactivity of the renin-angiotensin-aldosterone system (RAAS) plays a well-established role in the pathogenesis of hypertension [4].

Angiotensin-I converting enzyme (ACE) is a zinc metallopeptidase that is found in many tissues and biological fluids and it is an important player in regulating RAAS and kallikrein-kinnin system (KKS). There are two main mechanisms by which ACE causes hypertension. Firstly, ACE cleaves two amino acids from the decapeptide angiotensin I to produce the octapeptide angiotensin II, which is a strong vasoconstrictor. Secondly, ACE is involved in the degradation of bradykinnin, a potent vasodilator [5,6]. ACE is associated with hypertension and remains an important target for the discovery of anti-hypertensive drugs due to these effects. Currently, widely prescribed ACE inhibitors such as captopril and lisinopril are limited in their use due to well documented side effects such as cough, kidney problems, angioedema, and allergic reactions [7]. Furthermore, the increased incidence of cardiovascular diseases has spearheaded the search for safe and novel alternatives. Endogenous or biologically active peptides are options that are being explored due to their long-term regulation of tissue homeostasis, high bioavailability, and better safety profile [8,9,10].

Hemorphins are endogenous peptides that are produced from beta hemoglobin. Hemorphins of various lengths (4–10 amino acids) have been identified so far. These peptides are mainly produced in the plasma, brain, cerebrospinal fluid, and spinal cord [11,12,13,14]. The decapeptide LVV-hemorphin-7 (LVVYPWTQRF) is considered to be the longest form of hemorphin and is found abundantly in the mammalian nervous system. It is also the most stable and has the highest hydrophobicity when compared to other hemorphins [15,16]. Numerous studies have reported various biological activities of hemorphins, such as effects on spatial learning, analgesia, and hypertension [10,17,18,19,20]. Hemorphins have been shown to have beneficial effects in the control of blood pressure. The administration of hemorphin has also been shown to significantly reduce blood pressure in hypertensive rats [21]. Furthermore, studies have demonstrated that hemorphins produce anti-hypertensive effects by inhibiting ACE, which is a key component of RAAS [22,23,24].

The sequence of hemorphin peptide is highly conserved in mammals, which suggests similar biological activities in different mammals under different conditions. However, the camel hemorphin (LVVYPWTRRF) harbors a Q>R variation [25]. Camels have unique genetic adaptations that permit it to survive severe dehydration and very harsh environmental conditions [26,27]. Importantly, camel products, including its bioactive peptides, have already been reported in the treatment of various diseases [28,29,30]. In this study, we attempted to link the role of camel LVV-hemorphin-7 (LVVYPWTRRF) and hemorphin-7 (YPWTRRF) in the regulation of blood pressure with its likely direct action on ACE. The ACE inhibition potential of hemorphins from other closely related mammals (referred to as non-camel hemorphins here onwards) are well established. Based on this, we hypothesized that camel hemorphins may also target ACE and they could potentially produce better ACE inhibitory activities due to its unique variation near the C-terminus. In silico and in vitro methods were employed to investigate the molecular binding mechanisms and the ACE inhibition potential of hemorphins. Additionally, we examined the possible functional role of the three hydrophobic N-terminus residues (LVV-) by using shorter hemorphins without the LVV- sequence by employing extensive molecular docking and molecular dynamics studies.

## 2. Materials and Methods

### 2.1. Protein Structure Pre-Processing

The three-dimensional structure of ACE (PDB ID: 2XY9) was retrieved from RCSB Protein Data Bank (PDB) [31]. The retrieved protein structure was pre-processed using the Protein Preparation Wizard of Schrödinger Suite 2016-4 [32]. The protein preparation included the proper assignment of bond orders, adjustment of ionization states, orientation of disorientated groups, creation of disulphide bonds, removal of unwanted water molecules, metal and co-factors, capping of terminus amide, assignment of partial charges, and the addition of missing atoms and side chains. Hydrogen atoms were incorporated and standard protonation state at pH 7 was used. Finally, optimization and minimization were performed to obtain a geometrically stable structure [33].

### 2.2. Active Site Identification and Grid Generation

A receptor grid was generated, with default parameters, around the active site of the pre-processed protein while using the OPLS 2001 force field. For this, a van der Waal’s scaling factor of 1 and a charge cutoff of 0.25 was used. Finally, a cubic search space was generated, centered around the centroid of the active site residues of ACE.

### 2.3. Peptide Docking

Peptide docking was employed to predict the binding pose of hemorphins in ACE as well as to estimate the binding free energy. Standard precision (SP) flexible docking was performed using Schrödinger Glide version 2016-4 with appropriate penalties being applied for cis amide bonds [34]. The default parameters were used to dock the peptides. Flexible ligand sampling was used to generate various ligand conformations by the ConfGen algorithm [35]. The standard OPLS 2005 SP and post docking minimization was also performed in the gridded protein field. The poses were ranked based on the GlideScore (GScore) scoring function [36]. Finally, the lowest GScore value of the best docked pose was reported for each peptide.

### 2.4. Analysis of Docking Results and Binding Free Energy Calculation

Schrödinger Maestro was employed to visualize and interpret different types of interactions, including hydrogen bond interactions, hydrophobic interactions, and π interactions involving the best pose [37]. Binding free energy was calculated, in an implicit solvent model, using molecular mechanics-generalized Born surface area (MM-GBSA). Binding energy that was based on MM-GBSA was calculated using Schrödinger Prime employing the VSGB 2.0 implicit solvent model and the OPLS 2005 force field [38,39].

### 2.5. Molecular Dynamics (MD) Simulations

MD simulations were performed in triplicate to evaluate the dynamics and stability of the best binding pose of the LVVYPWTQRF, LVVYPWTRRF, YPWTQRF, and YPWTRRF peptides in the active site of ACE. MD simulations of the protein-peptide complexes were performed using Desmond employing the OPLS 2005 force field [40].

Four simulation systems—ACE with LVVYPWTQRF, ACE with LVVYPWTRRF, ACE with YPWTQRF, and ACE with YPWTRRF—were prepared. Three 200 ns simulations, each with a different set of initial velocities, were performed for each peptide. Single point charge (SPC) water model was employed to solvate all the systems in an orthorhombic box of water molecules, with a buffer distance of 10 Å [41]. The simulation system was neutralized and a 0.15 M NaCl salt concentration was maintained by adding a sufficient number of ions and counterions. The systems were subjected to the steepest descent minimization with Desmond’s default protocol prior to performing MD simulations. All of the systems were subjected to Desmond’s default eight stage relaxation protocol before the start of the 200 ns production run [42]. For the simulations, the isotropic Martyna-Tobias-Klein barostat and the Nose-Hoover thermostat were used for maintaining the pressure at 1 atm and temperature at 300 K, respectively [43,44]. Short-range and long-range coulombic interactions were evaluated with a cutoff of 9.0 Å using the short-range method and the smooth particle mesh Ewald method (PME), respectively [45]. A time-reversible reference system propagator algorithm (RESPA) integrator was employed with an inner time step of 2.0 fs and an outer time step 6.0 fs [46]. Data was saved to simulation trajectories every 100 ps. Finally, root mean square deviation (RMSD), root mean square fluctuation (RMSF), and protein-ligand contacts of the complexes were calculated from the trajectories.

### 2.6. In Vitro ACE Inhibition Assay

The inhibition of ACE was measured using the ACE Kit-WST (Dojindo Laboratories, Mashiki-machi, Japan). Custom synthesized hemorphin peptides (LVVYPWTQRF, LVVYPWTRRF, YPWTQRF, and YPWTRRF) were purchased from New England Peptide (Gardner, MA, USA). Indicator working solution and enzyme working solution were prepared according to the manufacturer’s instructions. 10 µL of each dose of hemorphin was added to the wells of a 96-well microplate. 20 µL and 40 µL of deionized water were added in blank 1 and blank 2 wells. 20 µL of the substrate buffer was added to each well, followed by 20 µL of enzyme working solution in all of the sample wells and blank 1. The plate was incubated for 1 h at 37 °C. After the incubation period, 200 µL of indicator working solution was added to each well and then incubated at room temperature for 5 min. After incubation, plate absorbance was measured at 450 nm using Glomax Discover Microplate Reader (Promega, Madison, WI, USA). The inhibition of ACE was calculated using the following equation: Inhibition rate (%) = (Ablank1 − Asample)/(Ablank1 − Ablank2) × 100.

From the obtained data, IC_50_ was calculated using Prism 7 (GraphPad, San Diego, CA, USA) from a non-linear regression plot of ACE inhibition (%) against peptide concentration (µM).

### 2.7. Statistical Analysis

Nonlinear regression was used to generate dose–response curves in Prism 7 (GraphPad, San Diego, CA, USA). The observed data are presented as mean ± SEM (Standard Error of the Mean). One-way or two-way analysis of variance (ANOVA), as appropriate, were carried out and a comparison of means was performed by Sidak’s multiple-comparisons test to estimate the statistical significance between the different treatments.

## 3. Results

### 3.1. Molecular Docking

The binding mode of non-camel hemorphins (LVVYPWTQRF and YPWTQRF) and camel hemorphins (LVVYPWTRRF and YPWTRRF) were determined using extensive docking and binding free energy calculations. The docked poses were evaluated with the GlideScore (GScore) scoring function [36] and the binding free energy for each binding mode was estimated while using the molecular mechanics-generalized Born surface area (MM-GBSA) method [38].

#### 3.1.1. Docking of Non-Camel and Camel LVV-Hemorphin-7 to ACE

Table 1 provides the binding score and amino acid residues of ACE that interacted with non-camel and camel LVV-hemorphin-7. The peptides docked in the S1, S2, and S1′ subsites of the active site of ACE. An extensive network of hydrogen bonds and hydrophobic and electrostatic interactions were formed between the hemorphins and ACE as shown in the 2D ligand interaction diagrams (Figure S1A,B).

**Figure 1:**
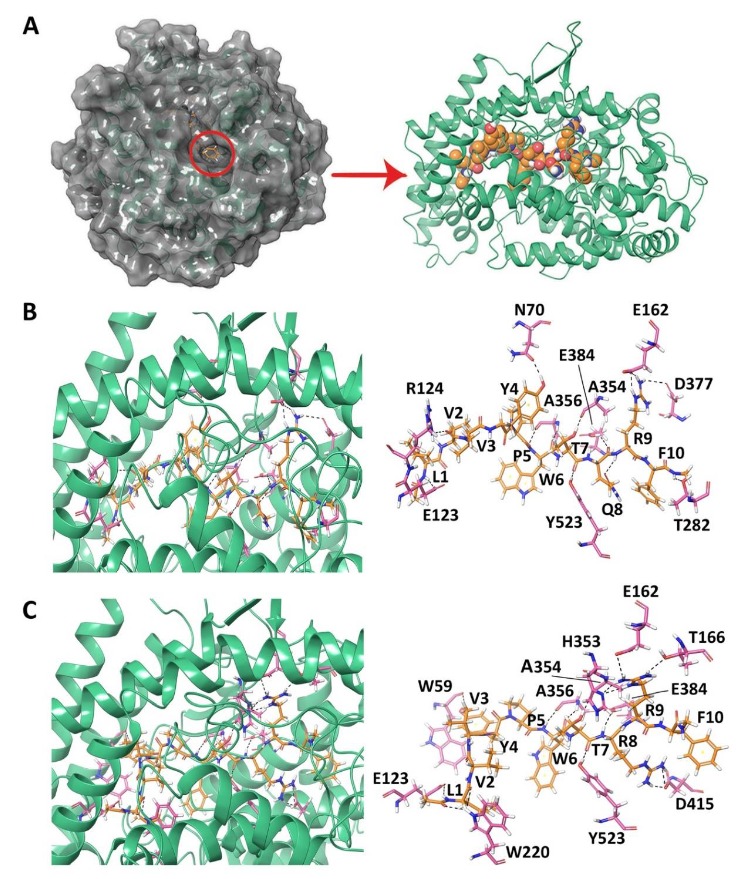
(**A**) Angiotensin-I converting enzyme with a peptide bound in the active site. (**B**) Docked pose and hydrogen bond interactions of LVVYPWTQRF with ACE. (**C**) Docked pose and hydrogen bond interactions of LVVYPWTRRF with ACE. The ACE protein is shown in green cartoon representation and its interacting residues are shown in pink stick representation; the docked ligand is represented as orange sticks, and hydrogen bonds are shown as black dashed lines.

The GScore and MM-GBSA binding energy that were obtained for the best binding pose of LVVYPWTQRF was −14.045 kcal/mol and −134.860 kcal/mol, respectively (Table 1). The sidechain amino group of LVV-hemorphin-7 residue Phe10 formed a hydrogen bond with Thr282 and hydrophobic interactions with Phe527 of ACE. It also exhibited a polar interaction with Gln281 in the S2 subsite and electrostatic interactions with Glu376, His383, and Asp453. The S1′ subsite residue Glu162 formed a hydrogen bond with the sidechain of Arg9. Additionally, Arg9 also showed hydrophobic interactions with Cys352, Val379, and Val380, and an electrostatic interaction with Lys454. Thr7, and Gln8 occupied the S1 and S2 subsites of ACE. The sidechain carboxyl group of Gln8 formed a hydrogen bond with Glu384, while the backbone hydroxyl and sidechain carboxyl groups of Thr7 formed hydrogen bonds with Ala354 and Tyr523, respectively. Gln8 also formed electrostatic and polar interactions with Glu411, Lys511, and His513 and hydrophobic interactions with Phe457, and Tyr520. His410 formed a cation-π interaction with Trp6. A hydrogen bond was also formed between the backbone amino group of Trp6 and Ala356. The residues Tyr4, Pro5, and Trp6 hydrophobically interacted with Leu139, Leu140, Trp357, Phe391, Tyr394, and Pro407, and electrostatically with Glu143, Asp358, Lys368, Glu403, and Arg522. The N-terminus residues (Leu1, Val2, and Val3) of LVVYPWTQRF formed hydrophobic interactions with Tyr51, Trp59, Tyr62, Ala63, Ile88, Ala89, Val119, and Leu122 of ACE. The sidechain amino group of Leu1 and sidechain carboxyl group of Val3 formed hydrogen bonds with Glu123 and Arg124, respectively (Figure 1B and Figure S1A).

The binding mode of LVVYPWTRRF was different from that of LVVYPWTQRF. The positioning of C-terminus residues (Arg8, Arg9, and Phe10) in the S1 and S2 binding pockets as well as the orientation of N-terminus residues were different. The best binding pose had a GScore of −18.824 kcal/mol and an MM-GBSA binding energy of −147.566 kcal/mol (Table 1). Phe10 bound in the S2 subsite and formed polar interactions with Asn277, Gln281, Thr282, and Asn285. It also showed electrostatic interactions with Glu376 and Asp453, and hydrophobic interactions with Val379 and Val380. Thr7, Arg8, and Arg9 residues of camel LVV-hemorphin-7 occupied the S1, S2, and S1′ binding pockets. A hydrogen bond was formed between the backbone amino groups of Arg9 and Glu162 in the S1′ subsite and with His353 in the S2 subsite, while the sidechain amino group formed a hydrogen bond with Thr166. It also formed hydrophobic interactions with Leu161 and Trp279. The Glu384 in S1 subsite and Asp415 showed hydrogen bonds with the backbone and sidechain amino groups of Arg8. It also showed electrostatic interactions with His383 and Glu411, and hydrophobic interactions with Phe457 and Phe527. The Thr7 residue of LVVYPWTRRF formed hydrogen bonds with Ala354 and Tyr523 in the S1 subsite. The Trp6 residue formed a hydrogen bond with Ala356 and cation-π interaction with His410. It also showed polar interactions with His387 and His513. Additionally, Pro5 and Trp6 produced electrostatic interactions with Trp357, Asp358, Tyr394, Glu403, and Pro407. The backbone hydroxyl group of Tyr4 exhibited a hydrogen bond with Trp59 and the sidechain formed π-π stacking with Tyr360. The Leu1 and Val2 residues of LVVYPWTRRF formed hydrogen bonds with Glu123 and Trp220, respectively. The first three residues of LVVYPWTRRF also showed hydrophobic interactions with Leu139, Ile204, Ala207, Met223, Val518, and Pro519, and an electrostatic interaction with Arg124 (Figure 1C and Figure S1B).

#### 3.1.2. Docking of Non-Camel and Camel Hemorphin-7 to ACE

**Figure 2:**
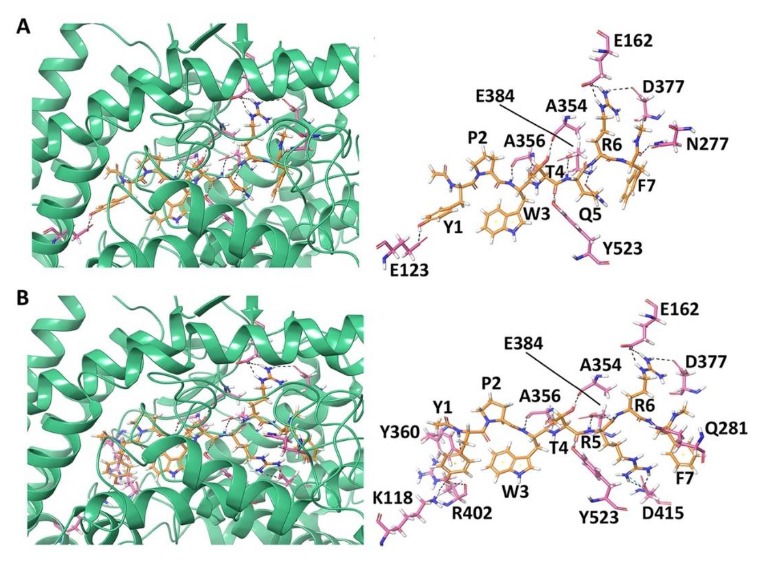
Docked pose and hydrogen bond interactions between hemorphin-7 and angiotensin-I converting enzyme (ACE) (**A**) YPWTQRF; and, (**B**) YPWTRRF. The ACE protein is shown in green cartoon representation and its interacting residues are shown in pink stick representation; the docked ligand is represented as orange sticks, and hydrogen bonds are shown as black dashed lines.

The best binding pose of non-camel hemorphin-7 (YPWTQRF) had a GScore of −15.955 kcal/mol and MM-GBSA binding free energy of −112.525 kcal/mol (Table 1). Tyr1 formed a hydrogen bond with Glu123 and π-π stacking with Trp59 of ACE. Tyr1 and Pro2 produced hydrophobic interactions with Tyr62, Ala63, Trp357, Tyr360, and Val380. The backbone amino and carboxyl groups of Trp3 formed hydrogen bonds with Ala356 and the sidechain formed a cation-π interaction with His410. Thr4 and Gln5 bound to the critical residues of S1 and S2 subsites. The backbone hydroxyl and sidechain amino groups of Thr4 formed hydrogen bonds with Ala354 and Tyr523 residues. The sidechain amino group of Gln5 formed a hydrogen bond with Glu384. Thr4 and Gln5 also formed hydrophobic interactions with Phe359, Tyr394, Phe457, Phe512, and Tyr520. The sidechain amino and carboxyl groups of Arg6 and Phe7 formed hydrogen bonds with Glu162, in the S1′ subsite, and Asn277, respectively. Arg6 also exhibited a hydrogen bond with Asn377. Arg6 formed polar interactions with Gln369, Thr371, and Thr372, and hydrophobic interactions with Cys352 and Cys370. Phe7 produced polar interactions with Thr166, Gln281, Thr282, and Asn285, and electrostatic interactions with Glu376, His383, Asp415, and Lys511 (Figure 2A and Figure S2A).

Camel hemorphin-7 (YPWTRRF) exhibited a different binding mode, especially the C-terminus residues (Arg5 and Phe7) and the N-terminus residues (Tyr1 and Pro2). Phe7 formed a hydrogen bond with Gln281 residue of S2 subsite. It also showed interactions with Thr282, Glu376, Asp453, Lys454, and Phe457. The sidechain amino groups of Arg6 formed hydrogen bonds with Glu162, in the S1′ subsite, and Asn377. It also produced polar interactions with Thr166, Asn285, Gln369, Thr371, and Thr372. The S1 subsite residue Glu384 and Asp415 produced hydrogen bonds with the sidechain of Arg5. It also formed a polar interaction with His353 in the S2 subsite and a hydrophobic interaction with Phe527. The Trp3 and Thr4 residues of camel YPWTRRF and non-camel YPWTQRF bound in a similar manner to ACE. Pro2 formed hydrophobic and polar interactions with Trp357, Val518, and Asn66. The sidechain hydroxyl and backbone amino groups of Tyr1 formed hydrogen bonds with Lys118, Tyr360, and Arg402. It also formed an electrostatic interaction with Glu403 and hydrophobic interactions with Tyr62, Ala63, Val399, and Phe512. Tyr1 also formed π-π stacking with Trp59 (Figure 2B and Figure S2B). The best binding pose had a GScore of −17.202 kcal/mol and a MM-GBSA binding energy of −130.392 kcal/mol (Table 1).

### 3.2. Molecular Dynamics Simulations

All-atom MD simulations were performed in triplicate using Desmond to evaluate the stability and dynamics of the interactions between ACE and hemorphins [40].

#### Simulations of Non-Camel and Camel Hemorphins Bound to ACE

**Figure 3:**
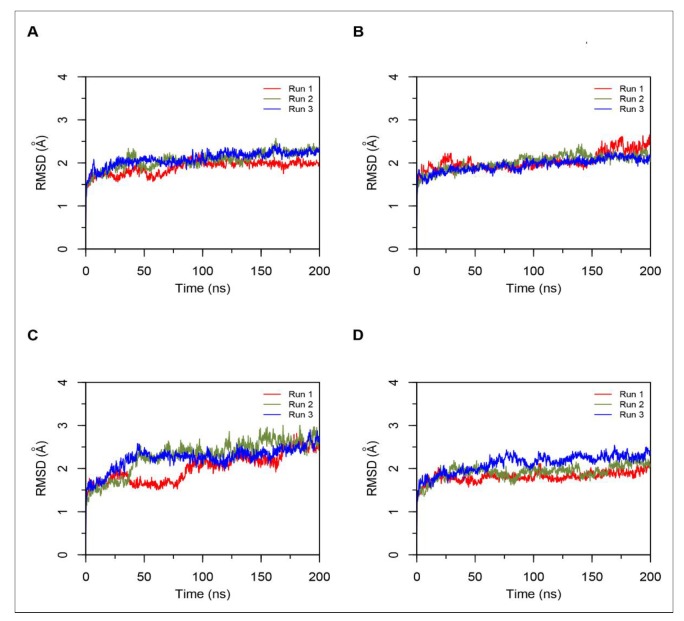
Root mean square standard deviation (RMSD) of protein Cα atoms obtained from three independent 200 ns runs of hemorphin bound ACE simulations. Data from the three runs are shown in red, green and blue. (**A**) Simulations of ACE-LVVYPWTQRF complex; (**B**) Simulations of ACE-LVVYPWTRRF complex; (**C**) Simulations of ACE-YPWTQRF complex; and, (**D**) Simulations of ACE-YPWTRRF complex.

**Figure 4:**
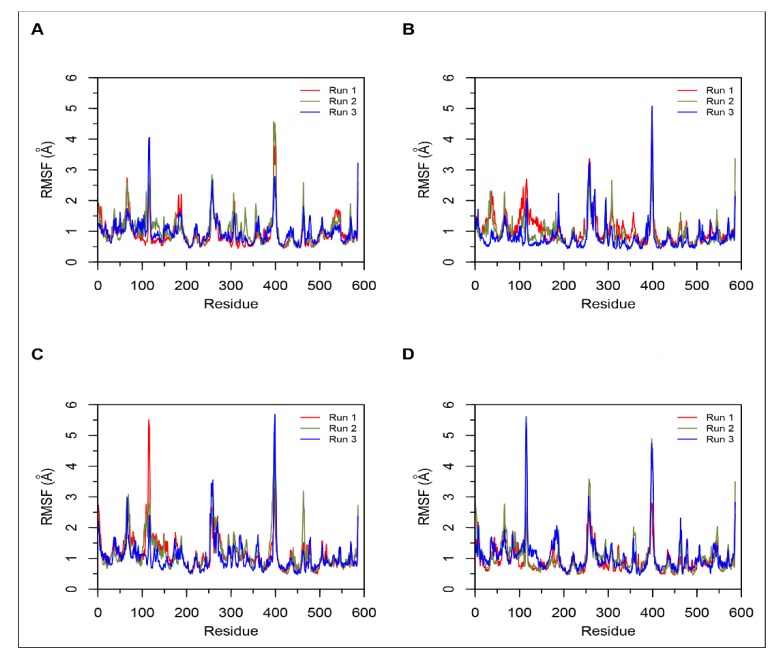
Root mean square fluctuation (RMSF) plots obtained from three independent 200 ns simulations of ACE. Data from the three runs are shown in red, green and blue. (**A**) Simulations of ACE-LVVYPWTQRF complex; (**B**) Simulations of ACE-LVVYPWTRRF complex; (**C**) Simulations of ACE-YPWTQRF complex; and, (**D**) Simulations of ACE-YPWTRRF complex.

**Figure 5:**
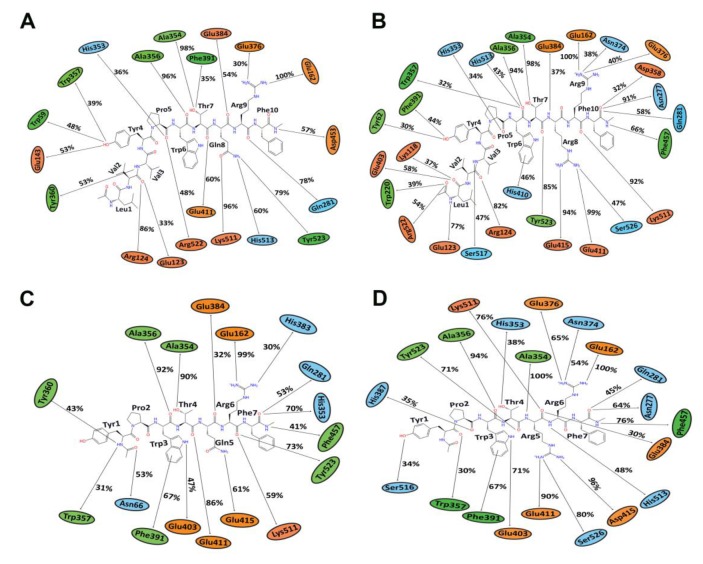
The average (from three runs) percentage of equilibrated simulation time during which various residues of hemorphin peptides interacted with residues of ACE. For this, the first 30 ns of each run was discarded. Orange, green, and blue colors represent charged, hydrophobic and polar amino acids, respectively. (**A**) Simulations of ACE-LVVYPWTQRF complex; (**B**) Simulations of ACE-LVVYPWTRRF complex; (**C**) Simulations of ACE-YPWTQRF complex; and, (**D**) Simulations of ACE-YPWTRRF complex.

MD results indicated that both LVVYPWTQRF and LVVYPWTRRF complexes remained stable throughout the duration of 200 ns simulations. The RMSD of ACE was around 2.0 Å for both LVV-hemorphin-7 bound structures (Figure 3A,B). In LVVYPWTQRF and LVVYPWTRRF bound simulations, most of the protein residues produced limited fluctuations, except for loop regions (Figure 4A,B), and the secondary structures were faithfully retained during the simulations. The C-terminus residues of LVVYPWTQRF were stabilized in S1, S2, and S1′ subsites by forming hydrogen and hydrophobic interactions. Glu162, Gln281, Glu384, Asp453, Lys511, His513, and Tyr523 maintained interactions with Gln8, Arg9, and Phe10 residues. The MD trajectory demonstrated that Arg9 interacted with Glu162, in the S1′ subsite, and Gln281, in the S2 subsite. Thr7 and Gln8 also occupied the S1 and S2 subsites and maintained sustained interactions with Ala354, Glu384, Lys511, His513, and Tyr523. The N-terminus residues of LVVYPWTQRF maintained interactions with Glu123, Arg124, and Tyr360. LVVYPWTQRF also formed interactions with Trp59, Glu143, His353, Glu411, Arg522, and Phe527. Similarly, the C-terminus residues of LVVYPWTRRF occupied S1, S2, and S1′ subsites and formed sustained interactions with the critical residues of these subsites. Arg9 consistently interacted with Glu162, in the S1′ subsite, Phe10 produced consistent interactions with Asn277, Gln281, and Phe457. Interestingly, the substituted Arg8 of LVVYPWTRRF peptide showed sustained interactions with Glu384, Glu411, Asp415, and Ser526, which were absent in the LVVYPWTQRF-bound simulations. Pro5, Trp6 and Thr7 formed sustained interactions with His353, Ala354, Ala356, His410, His513, and Tyr523. Additionally, the N-terminus residues of LVVYPWTRRF maintained interactions with Lys118, Trp220, Glu123, Arg124, Glu403, Ser517, and Arg522 throughout the simulation runs. Figure 5A, B show the average (from three runs) percentage of the equilibrated simulation time during which various residues of the two peptides interacted with residues of ACE.

Triplicate 200 ns simulations of YPWTQRF and YPWTRRF docked to ACE were also performed. In the YPWTQRF-bound simulations, the protein RMSD stabilized around 2.5 Å (Figure 3C). The secondary structure composition was conserved throughout the simulations and protein residues showed limited fluctuations apart from loop regions (Figure 4C). Arg6 and Phe7 were stabilized in the S1′ and S2 subsites, respectively, by forming sustained interactions with Glu162, Gln281, His353, Lys511, and Tyr523. Gln5 maintained interactions with Glu384 and Asp415 throughout the simulations. Thr4 interacted with S1 subsite residues Ala354 and Glu411. The N-terminus residues (Tyr1, Pro2, and Trp3) interacted with Asn66, Ala356, Trp357, Tyr360, and Glu403. Similarly, Arg6 and Phe7 residues of YPWTRRF occupied the S1′ and S2 subsites, respectively, by forming sustained interactions with Glu162, Gln281, and His513. They also maintained continuous interactions with Asn277, Asn374, Glu376, and Phe457. The S1′ pocket residue Glu162 interacted with Arg6 throughout the simulations. Interestingly, Arg5 of YPWTRRF maintained sustained interactions with Glu411, Asp415, Lys51, and Ser526 and with S1 subsite residues Ala354 and Glu384. Thr4 interacted with His353 and Tyr523. Additionally, the N-terminus residues (Tyr1, Pro2, and Trp3) showed continuous interactions with Ala356, Trp357, His387, Phe391, Glu403, and Ser516.

In YPWTRRF-bound simulations, the RMSD of the protein Cα atoms stabilized under 2 Å and the protein structure showed structural stability throughout the simulation (Figure 3D). The protein residues showed less fluctuations apart from loop regions (Figure 4D) and the secondary structure remained intact during the simulations. Figure 5C,D show the average (from three runs) percentage of equilibrated simulation time during which various residues of the two peptides interacted with residues of ACE.

### 3.3. In Vitro ACE Inhibition Assay

Molecular docking and MD simulation data suggested that camel LVV-hemorphin-7 and hemorphin-7 formed more hydrophobic and electrostatic interactions and hydrogen bonds with key residues in the active site that are important for ACE inhibition than non-camel LVV-hemorphin-7 and hemorphin-7, respectively. Based on these findings, we speculated that camel LVVYPWTRRF and YPWTRRF peptides could show lower IC_50_ than non-camel LVVYPWTQRF and YPWTQRF, respectively. To test this, we measured the ACE inhibition potential of both camel and non-camel peptides using an in vitro ACE inhibition assay.

#### 3.3.1. ACE Inhibitory Activity of Non-Camel and Camel Hemorphins

**Figure 6:**
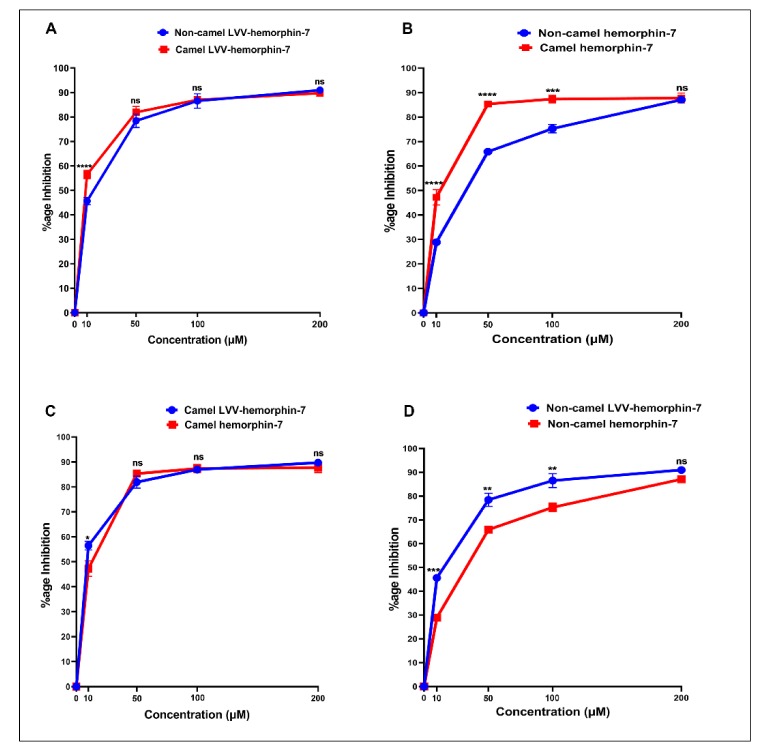
Dose-response curves of camel and non-camel hemorphins. ACE inhibition is shown in percentage for each dose. (**A**) Comparison of camel and non-camel LVV-hemorphin-7; (**B**) Comparison of camel and non-camel hemorphin-7; (**C**) Comparison of camel LVV-hemorphin-7 and hemorphin-7; (**D**) Comparison of non-camel LVV-hemorphin-7 and hemorphin-7. The comparison of different doses was done using two-way ANOVA and Sidak’s multiple-comparisons test to measure statistical significance between different hemorphins. Data are represented as mean ± SEM of three independent experiments. **** *p* < 0.0001, *** *p* < 0.001, ** *p* < 0.01, * *p* < 0.05 and ns *p* > 0.05.

**Figure 7:**
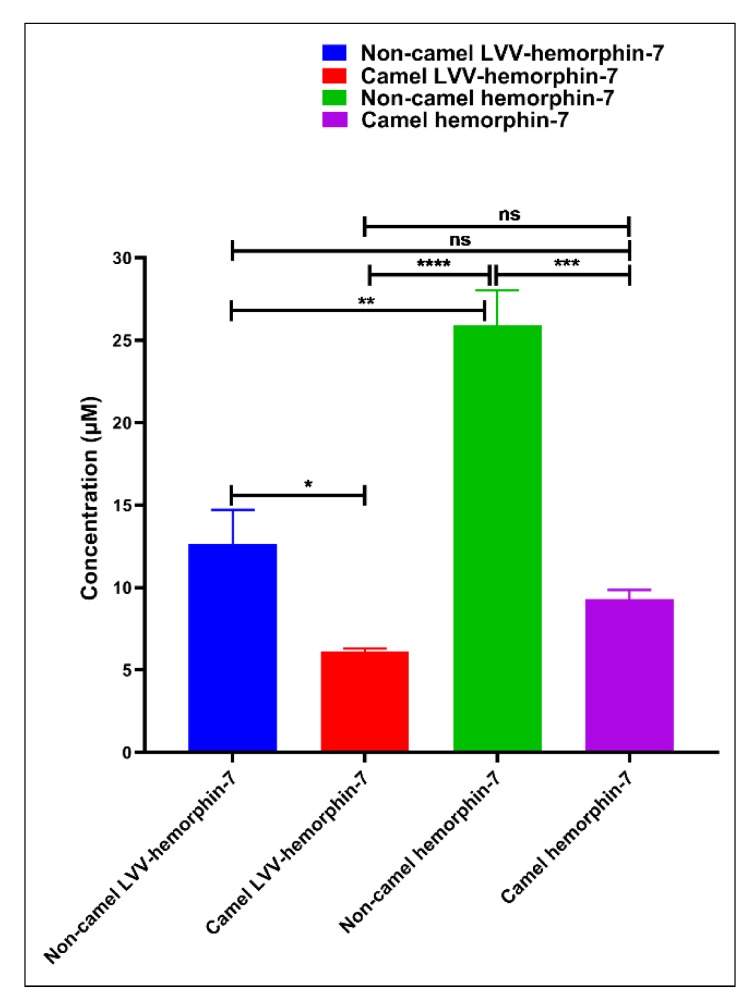
Half maximal inhibitory concentration (IC_50_) of both camel and non-camel hemorphins. IC_50_ values are expressed in micro molar (µM) units and comparisons of IC_50_ was done using one-way ANOVA and Sidak’s multiple-comparisons test to measure statistical significance between different hemorphins. Data are represented as mean ± SEM of three independent experiments. **** *p* < 0.0001, *** *p* < 0.001, ** p < 0.01, * *p* < 0.05, and ns *p* > 0.05.

The ACE inhibitory potential of both LVVYPWTQRF and LVVYPWTRRF peptides at different doses was evaluated. Both peptides showed ACE inhibitory activity at all tested doses (Figure 6A). LVVYPWTQRF and LVVYPWTRRF both exhibited reasonable saturation at 200 µM. Interestingly, the data showed a left shift in the dose-response curve of camel LVVYPWTRRF at all doses when compared to LVVYPWTQRF (Figure 6A). Importantly, this shift was significant (*p* < 0.0001, n = 3) at 10 µM, as shown in Figure 6A. Indeed, our further data sets confirmed the left shift of camel LVV-hemorphin-7. These results indicate a potentially more potent in vitro inhibitory action of camel-LVV-hemorphin-7 on ACE. LVVYPWTQRF and LVVYPWTRRF both exhibited a dose dependent inhibition of ACE with IC_50_ in the micromolar range (Figure 7). Interestingly, the IC_50_ of camel LVV-hemorphin-7 (6.601 µM) was significantly less than non-camel LVV-hemorphin-7 (12.649 µM) (*p* < 0.05, n = 3).

The ACE inhibitory activity of camel hemorphin-7 (YPWTRRF) and non-camel hemorphin-7 (YPWTQRF) was also measured at different doses. YPWTRRF produced a left shift at all doses in the dose response experiments when compared to YPWTQRF (Figure 6B). This shift was particularly significant at 10 µM, 50 µM, and 100 µM concentrations (*p* < 0.05, n = 3). The IC_50_ of camel hemorphin-7 (9.310 µM) was significantly lower than the non-camel hemorphin-7 (25.894 µM) (*p* < 0.001, n = 3), as shown in the (Figure 7).

#### 3.3.2. Comparison of the ACE Inhibition Potential of Camel and Non-Camel Hemorphins

In order to investigate the role of the first three N-terminus residues (Leu1, Val2, and Val3) of both LVVYPWTQRF and LVVYPWTRRF in ACE inhibition, we compared the ACE inhibition potential of LVVYPWTRRF with YPWTRRF and LVVYPWTQRF with YPWTQRF (Figure 6C,D). The IC_50_ calculations and comparisons showed that the IC_50_ of LVVYPWTRRF (6.601 µM) was lower than YPWTRRF (9.310 µM) (Figure 7).

On the other hand, a comparative analysis of the ACE inhibitory activity of LVVYPWTQRF and YPWTQRF showed that LVVYPWTQRF had significantly better ACE inhibition potential than YPWTQRF at 10 µM, 50 µM, and 100 µM (Figure 6D). IC_50_ of LVVYPWTQRF (12.649 µM) was also significantly lower than that of YPWTQRF (25.894 µM) (Figure 7). These results clearly suggested a positive role of the N-terminus residues (LVV-) of both LVVYPWTQRF and LVVYPWTRRF in the binding and inhibition of ACE.

## 4. Discussion

Hemorphins are a class of endogenous opioid peptides derived from hemoglobin. A number of studies have highlighted their therapeutic potential [10,17,18,19,47,48]. We had previously reported the molecular binding behavior of camel and non-camel LVV-hemorphin-7 on multiple targets [20,25]. Here, we report the binding and ACE inhibition potential of non-camel hemorphins (LVVYPWTQRF and YPWTQRF) and camel hemorphins (LVVYPWTRRF and YPWTRRF) using computational approaches and an in vitro ACE inhibition assay. Our findings demonstrate that both camel LVV-hemorphin-7 and hemorphin-7 bind more strongly to critical residues in the active site of ACE than non-camel LVV-hemorphin-7 and hemorphin-7, respectively. This finding is also supported by the in vitro ACE inhibition assay.

ACE is a membrane-bound zinc metallopeptidase that plays a vital role in blood pressure regulation by catalyzing the conversion of angiotensin I into angiotensin II, a potent vasoconstrictor [49]. ACE has a zinc ion binding motif, coordinated by His383, His387, and Glu411, which is important for the formation of the ACE-inhibitor complex. Furthermore, the active site of ACE is made up of three subsites, termed S1, S2, and S1′, which are also vital for ACE inhibition. These pockets harbor residues that interacts with potential inhibitors [6,50]. Ala354, Glu384, and Tyr523 constitute the S1 subsite; Gln281, His353, Lys511, His513, and Tyr520 constitute the S2 subsite, and Glu162 forms part of the S1′ subsite [51].

The binding of LVVYPWTQRF, LVVYPWTRRF, YPWTQRF, and YPWTRRF with ACE was elucidated using molecular modeling and simulations. Both camel and non-camel peptides bound stably in the active site of ACE. The C-terminus residues from position 7–10 of LVVYPWTRRF and LVVYPWTQRF, and residues from position 4–7 of YPWTRRF and YPWTQRF occupied all three active subsites of ACE (Figure 1 and Figure 2). The C-terminus residues (Arg9 and Phe10) of LVVYPWTQRF anchored the peptide in the S2 and S1′ subsites by forming hydrogen bonds with Glu162, Gln281, and Asp453. The Thr7 and Gln8 residues of LVVYPWTQRF occupied the S1 and S2 subsites by forming hydrogen bonds with Ala354, Lys511, His513, Tyr520, and Tyr523. These two residues of LVVYPWTQRF also interacted with Zn (II) ion and formed sustained interactions with His383, and Glu411 (Figure 1B). LVVYPWTRRF positioned itself in the active site of ACE slightly differently. LVVYPWTRRF was found to be deeply embedded in the active site. Arg9 and Phe10 stabilized the peptide by forming hydrogen bonds with Glu162, Asn277, Thr282, Asn374, and Glu376. They also formed polar interaction with Gln281 (Figure 1C). The Thr7 and Arg8 residues of LVVYPWTRRF formed sustained interactions with critical S1 and S2 subsite residues throughout the simulation runs. Additionally, Arg8 of LVVYPWTRRF also exhibited sustained interactions with Glu411, Asp415, and Ser526 than LVVYPWTQRF (Figure 5A,B). Non-camel hemorphin-7 and camel hemorphin-7 exhibited different binding modes. The positioning of N-terminus residues (Tyr1 and Pro2), and the C-terminus residues (Arg5, and Phe7) (Figure 2A,B). The YPWTRRF peptide exhibited strong interactions with almost all critical residues of ACE, including Glu162, Ala354, Ala356, His387, Glu411, Glu415, Lys511, and Tyr523 throughout the simulation runs, when compared to YPWTQRF (Figure 5C,D).

Clinically used ACE inhibitors captopril and lisinopril interact with Glu162, His353, Ala354, Glu384, His387, Glu411, Lys511, His513, Tyr520, and Tyr523 of ACE [52,53]. Enalaprilat, another well known ACE inhibitor, does not appear to interact with Glu162. This could be the reason why enalaprilat exhibited lower affinity when compared to lisinopril [53]. These amino acids are essential for designing potential ACE inhibitors. Among the hemorphins studied here, only camel hemorphin-7 interacted with His387. The other hemorphins interacted with all of the critical residues, other than His387 and Tyr520, in the active site of ACE.

Computational results indicated that camel LVV-hemorphin-7, and hemorphin-7 had better affinity when compared to non-camel LVV-hemorphin-7, and hemorphin-7, respectively. An ACE inhibition assay was performed to evaluate the IC_50_ of these peptides to investigate this further. Camel LVV-hemorphin-7 and hemorphin-7 had IC_50_ values of 6.601 µM and 9.310 µM, respectively (Figure 7). Notably, the IC_50_ of camel LVV-hemorphin-7 and hemorphin-7 were significantly lower than non-camel LVV-hemorphin-7 (12.649 µM) and hemorphin-7 (25.894 µM), respectively. These results clearly indicated that camel LVV-hemorphin-7 and hemorphin-7 exhibited more potent ACE inhibitory activity when compared to non-camel LVV-hemorphin-7 and hemorphin-7, respectively. However, the difference between LVVYPWTRRF and YPWTRRF was not significant (Figure 7). The results of non-camel hemorphins are comparable to previous studies that were performed to measure the ACE inhibitory activity of hemorphins [22,23]. Previous reports have indicated that LVVYPWTQRF has a higher ACE inhibitory activity than shorter hemorphins, such as YPWTQRF [54]. Their results demonstrated the importance of the hydrophobic N-terminus leucine-valine-valine residues for improved ACE inhibition. Additionally, the removal of the N-terminus leucine significantly decreased the ACE inhibition activity of VVYPWTQRF in comparison to LVVYPWTQRF. Another comparative study reported that LVVYPWTQRF was a more potent inhibitor of ACE than VVYPWTQRF [24]. These findings suggest that the N-terminus residues are important in ACE inhibition.

From a structure activity point of view of ACE inhibitors, studies have demonstrated that the ACE inhibitory activity of a peptide depends on the presence of specific residues at N and C-termini [8]. The presence of hydrophobic residues, particularly leucine, valine, isoleucine, and glycine, at the N-terminus of a peptide, produced higher ACE inhibitory activity, while aromatic amino acids, such as phenylalanine, proline, tryptophan, and tyrosine, are preferred at the C-terminus [55]. Additionally, the presence of positively charged residues, specifically arginine and lysine, near the C-terminus were also considered to be essential for the ACE inhibitory potential of peptides [56]. Moreover, this phenomenon has already been explained by numerous studies, which indicated that the ACE inhibitory potential of some peptides could be increased by the elongation of N-terminus residues [57,58,59]. Our in silico and in vitro results demonstrate that both camel and non-camel LVV-hemorphin-7 bound more stably to the active site of ACE and produced higher inhibition when compared to shorter hemorphin-7 peptides. The N-terminus residues (leucine-valine-valine) were observed to bind to residues outside the active site of ACE. This suggests that, in spite of the ACE inhibitory effect, this part of molecule might not have any direct interactions with the active site residues. The significantly improved ACE inhibitory activity of the peptides with the LVV- sequence could be due to the more stable binding of the peptide in the presence of this sequence. The comparative analysis of LVVYPWTRRF and LVVYPWTQRF showed that LVVYPWTRRF is a more potent ACE inhibitory peptide than LVVYPWTQRF (Figure 6A). Interestingly, the N-terminus residues of LVVYPWTRRF exhibited more sustained interactions with Tyr62, Lys118, Glu123, Arg124, Trp220, Glu403, Arg522, and Ser517, while an arginine substitution at position 8 formed more sustained interactions with Glu411, Glu415, and Ser526 when compared to LVVYPWTQRF (Figure 5A,B). This binding mode of the substituted arginine peptide showed that the N-terminus residues had been pulled deeper into the binding pocket. Thus, this could be the reason for the higher ACE inhibition potential of camel LVVYPWTRRF. These findings are in agreement with previous studies, which reported the importance of leucine and valine at the N-terminus and arginine near the C-terminus for improved ACE inhibition [54,56,58]. Additionally, the binding of peptide residues outside of the ACE active site, specifically with Tyr62, and Asn70 has also exhibited an important role in the non-competitive inhibition of ACE [60]. Moreover, a novel dual inhibitor occupying both the primary and secondary sites has also been reported [31]. Lys118, Glu123, Arg124, Trp220, Glu403, and Arg522 were considered to be the secondary binding site residues of ACE. This illustrates the plasticity of ACE active site in accommodating a diverse range of potent inhibitors [31]. A similar pattern was observed when LVVYPWTRRF was compared to the shorter camel hemorphin peptide (YPWTRRF) (Figure 5B,D). In the MD simulations, the N-terminus of LVVYPWTQRF exhibited sustained interactions with Glu123, Arg124, and Glu143 along with active site residues of ACE when compared to YPWTQRF (Figure 5A,C). Similarly, when the shorter camel (YPWTRRF) and non-camel (YPWTQRF) peptides were compared, YPWTRRF showed significantly lower IC_50_ and more sustained interactions with ACE active site residues than YPWTQRF (Figure 5C,D, and Figure 7). The additional interactions of the substituted arginine with Glu411, Asp415, and Ser526 anchored the peptide stably in the active site. Previous studies have already positively associated the presence of arginine near the C-terminus with higher ACE inhibition [56].

Overall, this is the first study reporting the comparative ACE inhibition potential of camel hemorphins and non-camel hemorphins. Interestingly, camel hemorphin is unique due to a Q>R amino acid substitution near the C-terminus, when compared to other closely related mammals. The in silico and in vitro results showed that camel hemorphins produced more sustained interactions, with critical residues of ACE, and exhibited lower IC_50_ when compared to non-camel hemorphins. It would be worth exploring the action of camel hemorphins on other important players of RAAS, such as angiotensin II type I receptor, bradykinin, and vasopressin receptors, which are known to play a prominent role in regulating blood pressure and the cardiovascular system [61,62].

## Figures and Tables

**Table 1:** Interactions of the best binding pose of LVVYPWTQRF, LVVYPWTRRF, YPWTQRF, and YPWTRRF peptides with angiotensin-I converting enzyme (ACE).

Peptide	Glide Docking Score-GScore (kcal/mol)	MM-GBSA (kcal/mol)	Residues Forming Hydrogen Bonds	Residues Forming Hydrophobic Interactions	Residues Forming π-π Stacking or Cation-π Interactions
LVVYPWTQRF	−14.045	−134.860	Asn70, Glu123, Arg124, Glu162, Thr282, Ala354, Ala356, Glu384, Tyr523	Tyr51, Trp59, Tyr62, Ala63, Ile88, Ala89, Val119, Leu122, Ala125, Leu139, Leu140, Cys352, Trp357, Tyr360, Cys370, Val379, Val380, Phe391, Tyr394, Ala418, Phe457, Phe512, Val518, Tyr520, Phe527	His410
LVVYPWTRRF	−18.824	−147.566	Trp59, Glu123, Thr166, Glu162, Trp220, Ala354, Ala356, Glu384, Asp415, Tyr523	Tyr62, Ala63, Ile88, Leu139, Leu161, Ala204, Ala207, Ala216, Met223, Trp279, Val379, Val380, Phe391, Tyr394, Pro407, Phe457, Phe512, Val518, Pro519, Phe527	Tyr360, His410
YPWTQRF	−15.955	−112.525	Glu123, Glu162, Asn277, Ala354, Ala356, Asn377, Glu384	Tyr62, Ala63, Trp279, Cys352, Trp357, Phe359, Tyr360, Cys370, Val379, Val380, Phe391, Tyr394, Pro407, Phe457, Phe512, Tyr520, Phe527	Trp59, His410
YPWTRRF	−17.202	−130.392	Lys118, Glu162, Gln281, Ala354, Ala356, Tyr360, Asn377, Glu384, Arg402, Asp415, Tyr523	Tyr62, Ala63, Ile88, Trp279, Trp357, Cys370, Val379, Val380, Phe391, Tyr394, Pro407, Phe457, Phe512, Val518, Phe527	Trp59, His410

## Supplementary Materials

The following are available online at https://www.mdpi.com/2218-273X/10/3/486/s1, Figure S1: Interactions of LVV-hemorphin-7 with ACE, Figure S2: Interactions of hemorphin-7 with ACE.
