# Supplementary material for: Camel Hemorphins Exhibit a More Potent Angiotensin-I Converting Enzyme Inhibitory Activity than Other Mammalian Hemorphins: An In Silico and In Vitro Study
Source: Biomolecules. 2020 Mar 23;10(3):486. doi: 10.3390/biom10030486 (PMC7175181; doi:10.3390/biom10030486)
Supplement: Supplementary file 1 [file biomolecules-10-00486-s001.pdf]

Camel hemorphins exhibit a more potent angiotensin-I converting enzyme inhibitory activity than other mammalian hemorphins: An *in silico* and *in vitro* study

Amanat Ali[1], Seham Rashed Ali Alzeyoudi[1], Shamma Saleh Almutawa[1], Alya Nasir Alnajjar Banialnajjar[1], Yusra Al Dhaheri[1] and Ranjit Vijayan[1]*

[1]Department of Biology, College of Science, United Arab Emirates University, PO Box 15551, Al Ain, Abu Dhabi, United Arab Emirates.

*Correspondence: Tel: +971 3 713 6302, Email: ranjit.v@uaeu.ac.ae

Supplementary Materials

A

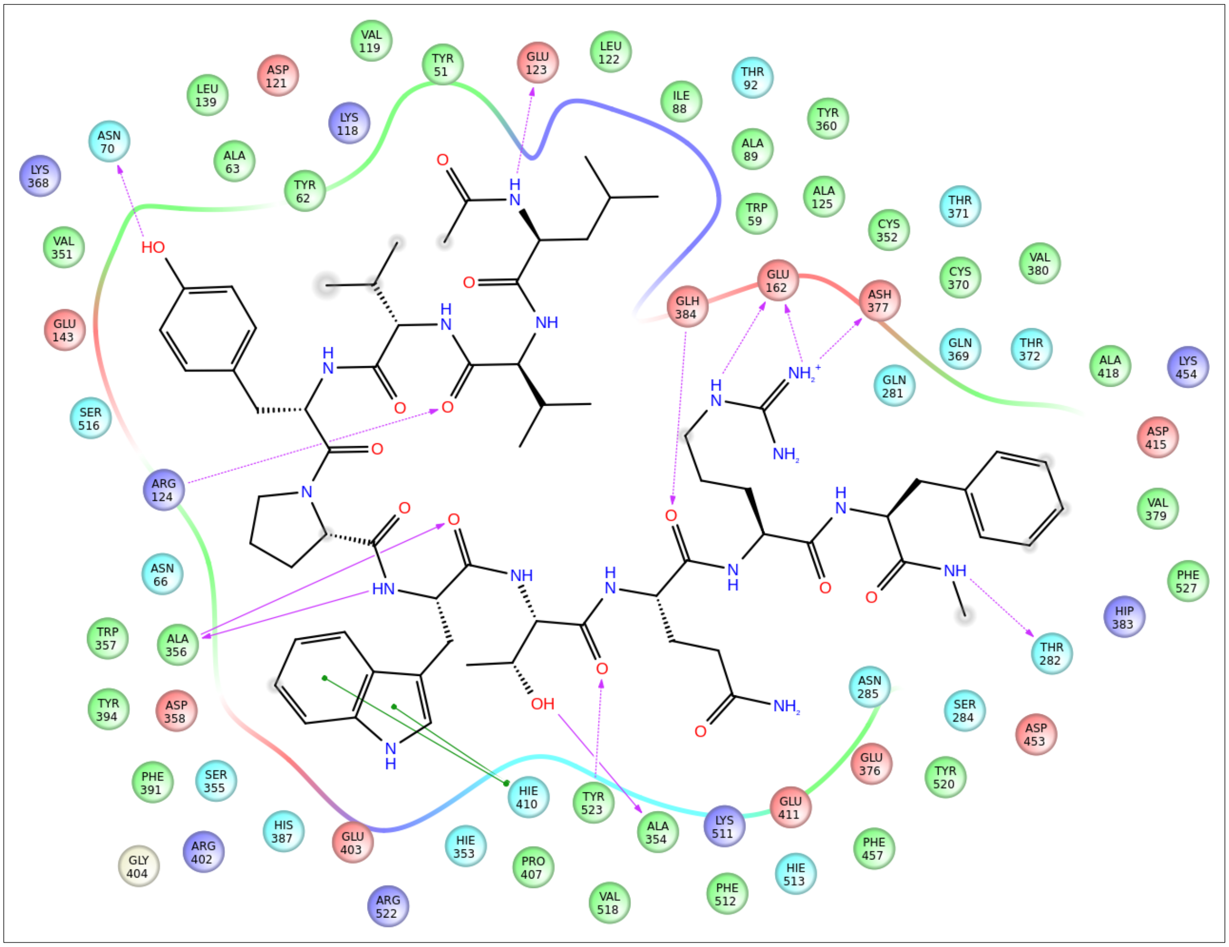

B

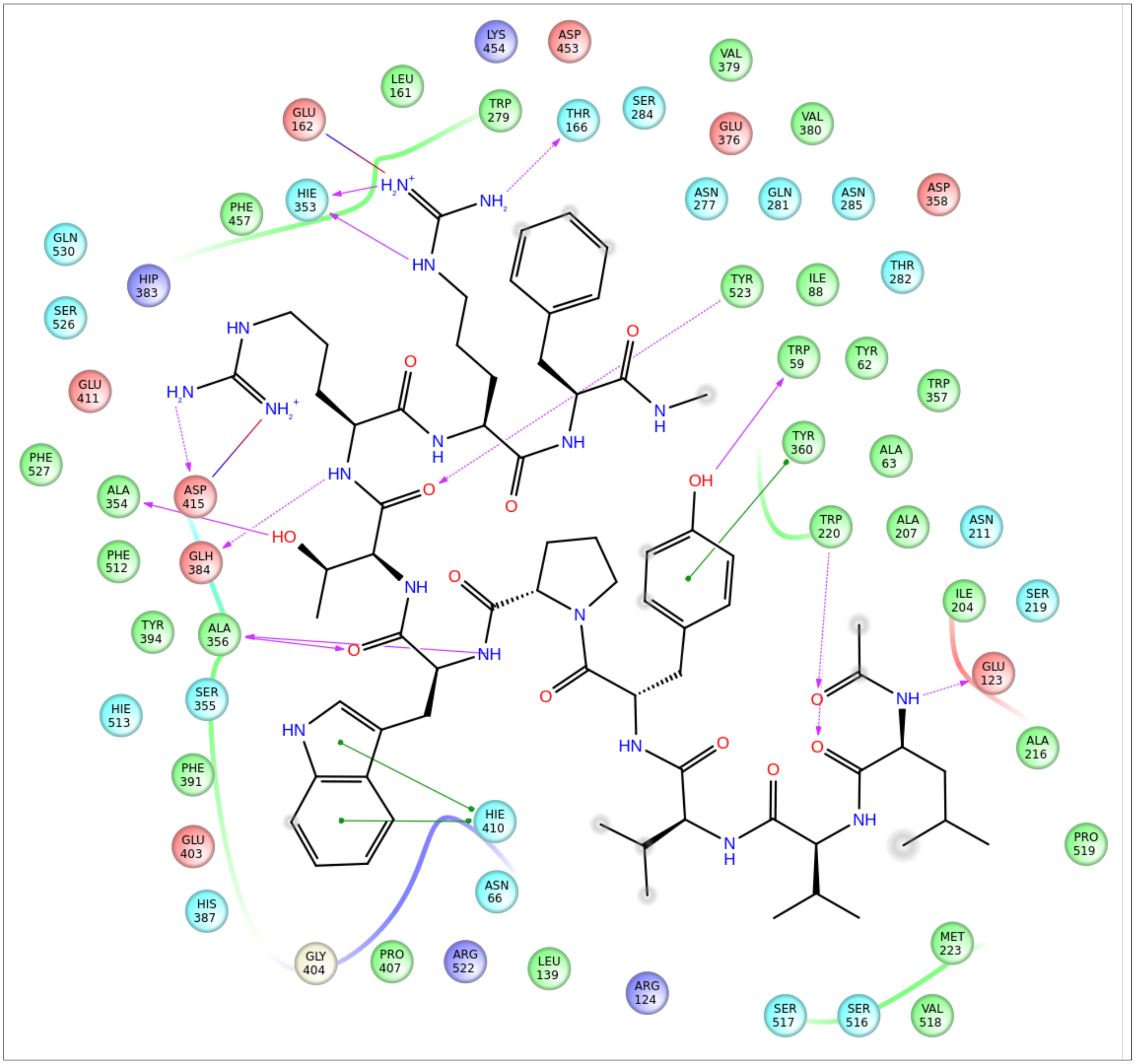

Figure S1. Interactions of LVV-hemorphin-7 with ACE. A) ACE-LVVYPWTQRF. B) ACE-LVVYPWTRRF.

A

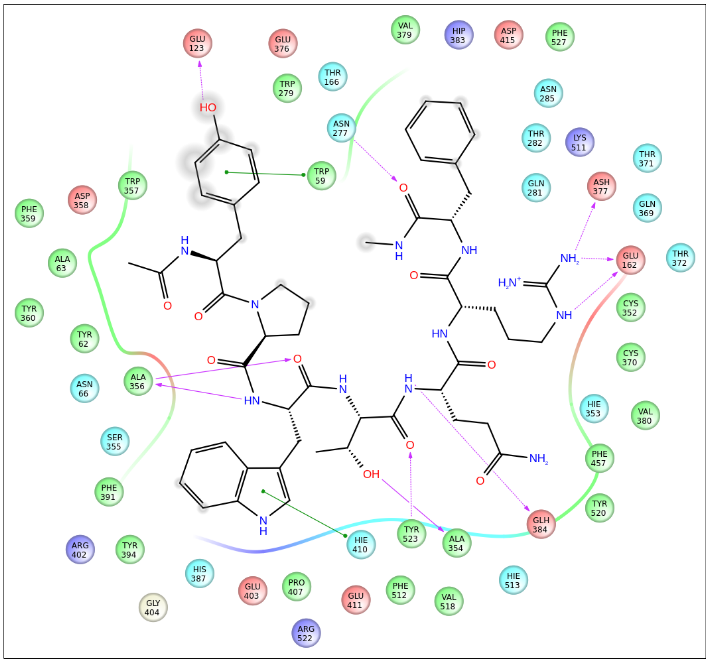

B

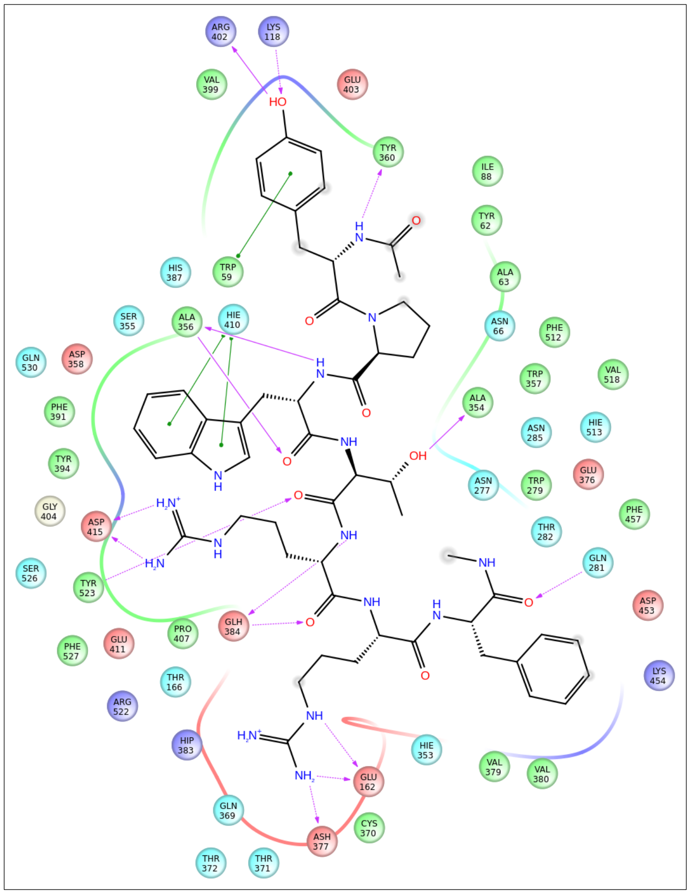

Figure S2. Interactions of hemorphin-7 with ACE. A) ACE-YPWTQRF. B) ACE-YPWTRRF
